# Forming Analysis and Heat Treatment of TC31 Titanium Alloy Component with High Ribs and Thin Webs

**DOI:** 10.3390/ma16072860

**Published:** 2023-04-03

**Authors:** Heping Deng, Wu Min, Anjun Mo, Yi Qin, Shixin Peng, Fanjiao Gongye, Shishan Li, Jie Zhou

**Affiliations:** 1 Chongqing Key Laboratory of Advanced Mold Intelligent Manufacturing, College of Materials Science and Engineering, Chongqing University, Chongqing 400044, China; 2 China National Erzhong Group, Wanhang Die Forging Co., Ltd., Deyang 618013, China; 3 Chongqing Jiepin Technology Co., Ltd., Chongqing 400050, China; 4 Chongqing For-Green Technology Co., Ltd., Chongqing 400044, China

**Keywords:** TC31 titanium alloy, high ribs and thin webs, forging forming, heat treatment, mechanical properties

## Abstract

TC31 is a new type of high-temperature titanium alloy, but few researchers have studied the combination of forming and heat treatment of a component using this material. The component with high ribs and thin webs was studied by numerical simulation and trail production. Based on the establishment of the finite element model, the forming process was analyzed by simulation software, and the maximum forming load of the component was 1920 kN. Ultimately, there were no folding defects of the component during the forming process. The material flow law was revealed by selecting the typical section of the component, and then the forming process was verified and the fully filled component was obtained. After that, the component was subjected to post-processing, and three heat treatment methods were designed to conduct heat treatment experiments on it (heat treatment: solution treatment and aging treatment). By analyzing the influence of three heat treatment methods on mechanical properties, the optimal heat treatment method was obtained, namely a solution treatment at 960 °C for 2.5 h and aging treatment at 610 °C for 7 h. The ultimate tensile strength, yield strength, elongation, and section shrinkage of the component through forging forming and heat treatment are higher than those of original material; meanwhile, it also indicates that the designed heat treatment has a better effect on the high-temperature mechanical properties of this titanium alloy at 650 °C than that at 450 °C. The research on the combination of the forming and heat treatment of this component provides a reference for the engineering application of high-temperature titanium alloys.

## 1. Introduction

Titanium alloy has the characteristics of high specific strength, high-temperature resistance, and strong corrosion resistance, so it is widely used to form key structural components in the aerospace industry [1,2,3]. Among all titanium alloys, high-temperature titanium alloys are mainly used in the aerospace industry. TC31 titanium alloy, a new high-temperature dual-phase (α + β) titanium alloy of the Ti-Al-Sn-Zr-Nb-Mo-W-Si system, can be used in a temperature range from 650 °C to 700 °C [4,5]. Because the alloy has good durability and creep property under high load and high temperature, it has shown good application in aerospace field [6,7,8]. However, titanium alloys have high strength and low ductility at room temperature, so they usually need to be heated to form [9,10]. Hot working is the most applicable forming process for the alloys [11]. Hot forging, a forming technology, improves mechanical properties without sacrificing the complex shape of the components through transforming the coarse heterogeneous microstructure into a fine and homogeneous equiaxial microstructure [12,13]. The forging process plays a great role in the forming of structural titanium alloy components [14]. Hot forging is also a nonstationary and complex forming process, which includes billet heat transfer (with dies and environment), billet flow and filling, etc. [15]. To obtain an excellent component of high-temperature titanium alloy, the hot forming process has become one of the key points.

In order to reveal the characteristics of components during the forming process, the method of finite element simulation has been focused on by many researchers. Tong [16] simulated the forging of TA15 alloy rods by using the finite element method (FEM), and the results provided a significant theoretical foundation for the practical production. Zhu et al. [17] also studied the influence of deformation and the initial forming temperature on the microstructure of titanium alloy by FEM. Moreover, the interaction between the deformation behavior and microstructure evolution during titanium alloy forging was studied by FEM [18]. Zhang et al. [19] used a combination of finite element and experimental methods to investigate the influence of friction factors on the forming of TA15 alloy rings. However, few researchers have studied the forming of TC31 titanium alloy components.

A component can be successfully applied in engineering. Besides its forming process, the effects of post-processing on its performance should be considered. Heat treatment is one of the important post-processing technologies, as it plays an important role in improving the mechanical properties of alloy [20,21,22]. Cheng et al. [23] studied the effects of heat treatment on the mechanical properties of a novel high-temperature titanium alloy (Ti55). Yu et al. [24] investigated a three-stage heat treatment method for Ti6Al4V alloy and revealed the effects of microstructure on strength and ductility. Wang et al. [25] investigated the formation and characteristics of bilamellar microstructure in Ti6242S titanium alloy under a dual heat treatment (single-phase heat treatment + two-phase heat treatment). Moreover, some researchers have demonstrated that the mechanical properties of titanium alloys largely depend on the morphology, distribution, and size of the α phase [26,27,28]. Zhu et al. [29] studied the effects of heat treatment on the microstructure of titanium alloy (Ti-5Al-2Sn-2Zr-4Mo-4Cr). However, there are few reports about the heat treatment of TC31 high-temperature titanium alloy, especially for an engineering application component.

Taking the cue of above viewpoints, this work aims to investigate a TC31 titanium alloy component with high ribs and thin webs. On the basis of establishing the finite element model, the forming load, filling, and material flow of the typical section of the component were analyzed by simulation software, and then the forming process was verified by trial production. Three heat treatment methods were designed for the trail-produced component. The component was first subjected to different heat treatments, which were the followed by mechanical properties tests and microstructure observations. Finally, the characteristics of the microstructures under different heat treatments were analyzed and compared. This work can provide some guidance for the forming and heat treatment methods of TC31 high-temperature titanium alloy, so the research can also promote the engineering application of this alloy.

## 2. Component and Forming Analysis

### 2.1. Component and Its Material

**Figure 1:**
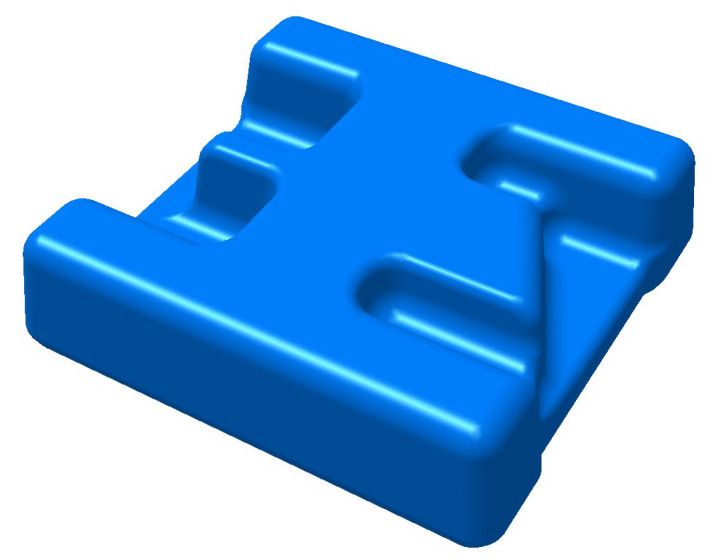
Three-dimensional model of the component.

**Figure 2:**
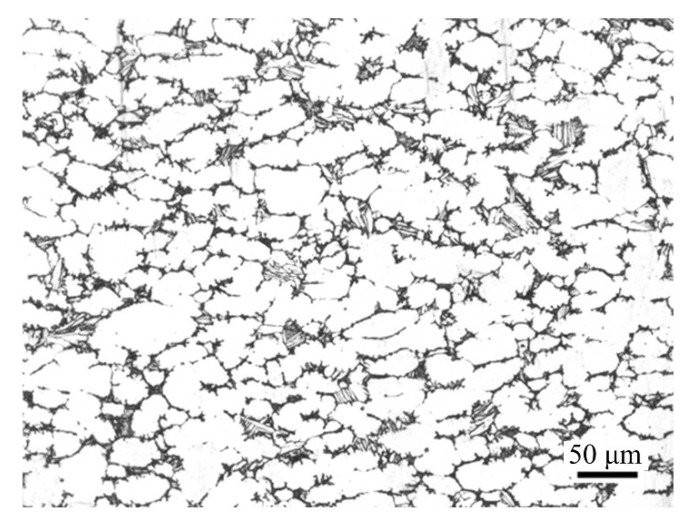
Microstructure of the as-received material.

The three-dimensional model of the component is shown in Figure 1. It is composed of high ribs of different shapes and webs with stepped shapes. The size of its outline is 200 mm × 200 mm × 75 mm. The material of the component is TC31 titanium alloy, which belongs to α + β type high-temperature titanium alloy in this paper. The composition and mechanical properties of the material are listed in Table 1 and Table 2, respectively. The microstructure of the as-received material is shown in Figure 2.

### 2.2. Forming Design

**Figure 3:**
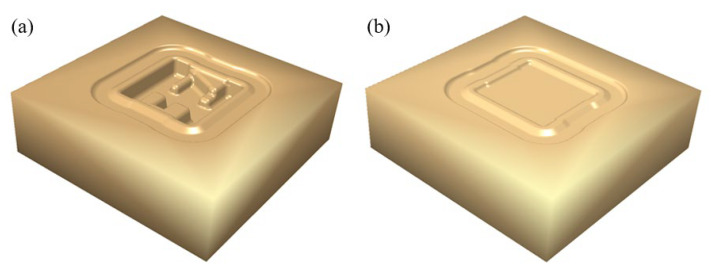
The designed structure of dies: (**a**) top die and (**b**) bottom die.

In view of the structural characteristics of the component, it has a typical high rib and thin web. The hot forging process was used to form the component, and this process not only saved material but also ensured its performance. In order to enable the structures of dies where the high ribs of the component correspond to be filled smoothly, the side with a complex structure was selected as the top die, and the other side was the bottom die. The designed structure of dies is shown in Figure 3.

## 3. Finite Element Analysis and Verification

### 3.1. FE Modeling

**Figure 4:**
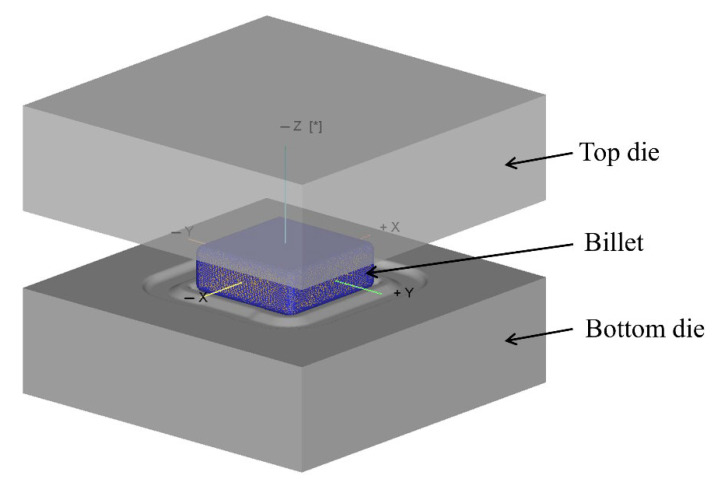
FE model of forming: [*] represents the direction of movement.

In order to study the laws of the forming process of the component, the finite element (FE) method was used to simulate the process. The finite element (FE) model is shown in Figure 4. In the simulation process, the top die and bottom die were regarded as rigid bodies, and the billet was regarded as the plastic body because the focus was on the forming of the component. The billet was divided as tetrahedron mesh, the friction constant coefficient of the forming process was assumed to be 0.3, and the heat transfer coefficient between the billet and dies was 5 N/s/mm/°C [30]. The main process parameters of finite element simulation are listed in Table 3.

### 3.2. Simulation of Forming Process

**Figure 5:**
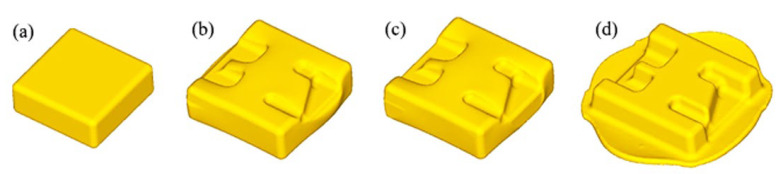
Schematic diagram of forming process: (**a**) blanking, (**b**) pre-forging, (**c**) trimming, and (**d**) final forging.

The main forming process of the component included blanking (square billet), pre-forging, trimming, and final forging, as shown in Figure 5. The filling condition and load of the component with high ribs and thin webs under the specific production process were studied by finite element simulation. The pre-forging was performed with a remaining 20 mm forging stroke. After the pre-forging, the flash edge of component was trimmed and heated to 975 °C, and then the final forging was performed with a remaining 4 mm forging stroke.

**Figure 6:**
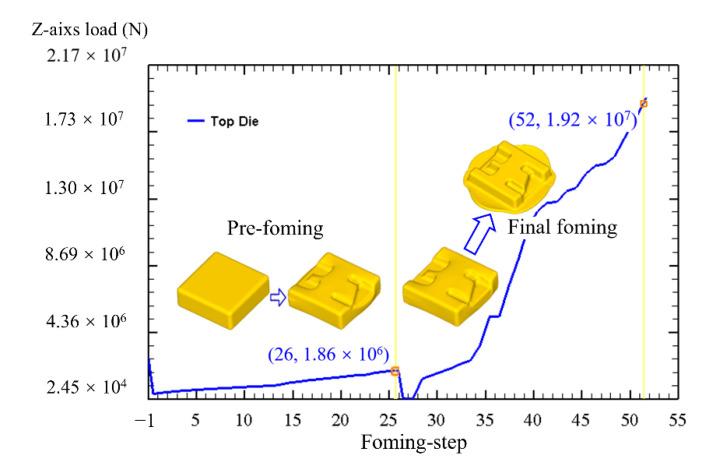
Forming load curve of the TC31 component.

In the finite element model, as shown in Figure 4, the bottom die was fixed, the top die moved downward, and the billet was forced to be filled into each structure under the action of the top die. The movement direction of the top die was +Z-axis direction under the custom coordinates, and the load during the forging forming process can be expressed by the following formula:

(1) $$F=Max\left(F_{z}\right)$$

where *F* is the maximum load, $F_{z}$ is the forming load corresponding to the die moving along the *Z* axis (vertical direction), and the unit of *F* is N. The load curve of the forging forming process is shown in Figure 6. The incremental steps from step −1 to step 26 corresponding to pre-forging, the incremental steps from step 26 to step 52 corresponding to final forming. As the pre-forging process progressed, the load increased gradually. When the forging die retained a stroke of 20 mm (the incremental step corresponded to 26th step), the pre-forging load reached the maximum value of 186 kN (186 t). During the final forging process, the load curve increased faster and higher, and it reached the maximum value of 1920 kN (1920 t) when the forging die retained a stroke of 4 mm (the incremental step corresponded to 52th step). Therefore, the forming loads of the pre-forging and final forging of the component are 186 t and 1920 t, respectively.

**Figure 7:**
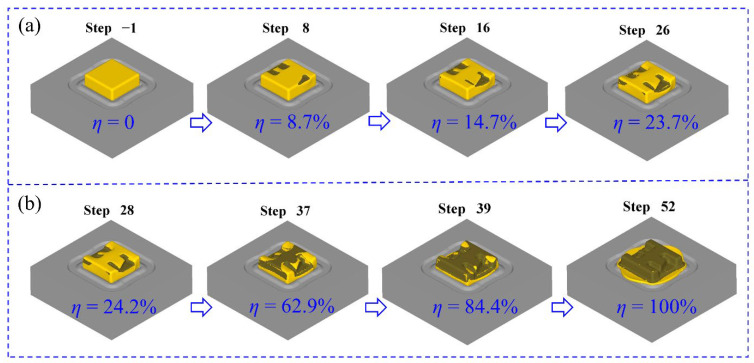
The main forming process of the component: (**a**) pre-forging and (**b**) final forging.

In order to investigate the filling law of materials during the forming process, the filling rate was used to represent the filling of materials with different die closure heights. In other words, the filling rate can also roughly indicate the forming rate of the component itself, and then the forming condition of each rib can also be analyzed. The filling rate can be expressed by the following formula [31]:

(2) $$\eta =\frac{S_{1}}{S_{0}}\times 100\%$$

where *η* is the filling rate of the forging, $s_{1}$ is the actual contact area between the die and the billet, and $s_{0}$ is the ideal contact area between the die and the billet. Considering the structural characteristics of the dies in Figure 3, the complex shape features of the component are mainly focused on the top die side, so the top die is mainly used for forming the component, and the bottom die mainly plays a role in positioning the billet. Therefore, the filling rate of the component mainly depends on the contact area between the top die and the billet. Figure 7 shows the main forming process of the component. During the pre-forging forming process, the filling rate increases gradually. With the increase of the incremental step, the filling rate increases relatively uniformly. During the process of final forging, the filling rate in the early stage increases relatively faster, and the increase in the later stage is relatively small. It can be seen from Figure 6 that the change of the filling rate in the whole forming process has a good match with the change of load. For example, the filling rate changed by 21.5% between the 37th and 39th incremental steps, showing that the contact area between the billet and the dies suddenly increased, and the billet was forced into the cavity of the dies; the load curve corresponding to the incremental steps also shows a sharp change.

### 3.3. Assessment of Folding Risk

**Figure 8:**
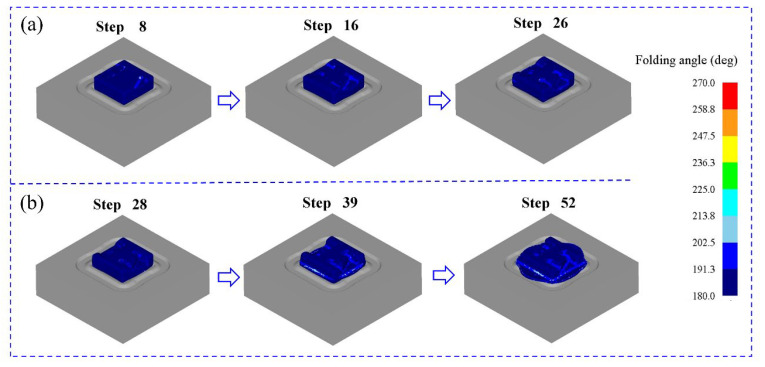
Distribution of folding angles during forming: (**a**) pre-forging and (**b**) final forging.

In order to investigate whether there were some defects of folding during the forming process, the folding-angle method was used to evaluate the whole process. It can be seen from Figure 8 that the change of the folding angle is relatively small during the pre-forging process. During the final forging process, the change of the folding angle is larger than that of the pre-forging, and it is mainly distributed on the flash edge of the component. By analyzing the whole forming process, we see that the changing ranges of the folding angle are relatively small, and they are all distributed in a range from 180° to 210°, which reveals that there are no folding defects in the filling process of the material. Therefore, the rationality of the die structure is further proved.

### 3.4. Flow Analysis of Materials

**Figure 9:**
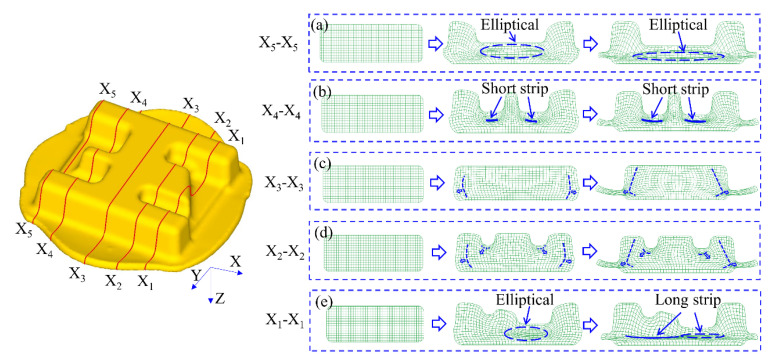
Flow of materials with different sections: (**a**) X_5_-X_5_, (**b**) X_4_-X_4_, (**c**) X_3_-X_3_, (**d**) X_2_-X_2_, and (**e**) X_1_-X_1_.

To meet the needs of the analysis, the forming process was divided into the beginning stage, the middle stage, and the final stage. The flow of material in different sections of the component was different. The sections which are parallel to XY plane were selected to cut it. The five typical sections were X_1_-X_1_, X_2_-X_2_, X_3_-X_3_, X_4_-X_4_, and X_5_-X_5_, as shown in Figure 9. As the forming process proceeded, the top die gradually contacted the billet, and then the billet was brought into the die cavity. The corresponding cross-section in Figure 9a is X_5_-X_5_. In the middle stage of forming, the mesh in the middle of the billet is compressed in the Z-axis direction to form an elliptical shape, while the materials on both sides flowed outward to form a burr (flash). In the final stage of forming, the flow tendency is more obvious, and the elliptical shape is further compressed. The corresponding section in Figure 9b is X_4_-X_4_. In the middle stage of forming, the mesh on both sides of the middle rib is compressed to form a short strip, till the final stage of forming, when it is compressed to a more obvious short strip. The corresponding section in Figure 9c is X_3_-X_3_. During the middle and final stages of forming, the material flow mainly flows toward the burr part because the section has no rib structure. The corresponding section in Figure 9d is X_2_-X_2_. Because there is a wide rib in the section, the mesh on both sides of the middle rib is compressed less obviously than that of section X_4_-X_4_, and the flow of the outermost material is similar to section X_3_-X_3_. The corresponding cross-section in Figure 9e is X_1_-X_1_. In the middle stage, the material flow forms an elliptical shape. Once the forming stage progresses, the elliptical shape is further compressed, and the surrounding mesh forms a long strip together.

**Figure 10:**
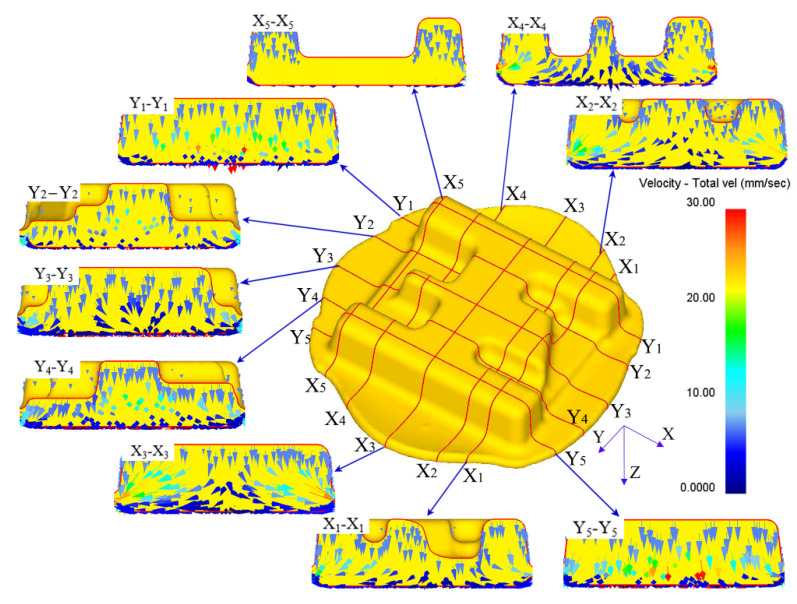
Velocity field distribution of different sections.

Due to the inertia of the billet, the material in contact with the top die first flows, and the material that contacts the bottom one flows slowly. To some extent, it indicates that the material in contact with the latter will also be driven by the first flowing material. As shown in Figure 10, the speed of the material on the top die side of each section of the whole forging is faster than that on the bottom die side. Therefore, it also provides a reference for the design of a similar high-rib component. The part that is difficult to fill should correspond to the side where the material easily flows, and the surrounding materials should have a complementary effect on its filling. A good streamline distribution is the basis for it to obtain good comprehensive mechanical properties, so it is helpful to form a good streamline distribution by understanding the material flow of a component.

### 3.5. Verification of Forming

**Figure 11:**
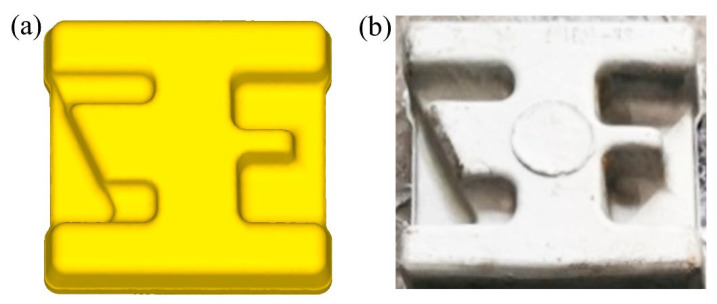
Comparison of components: (**a**) simulated component and (**b**) trial-produced component.

The numerical simulation analysis was verified by the forging experiment. The trial production component was obtained as shown in Figure 11. It can be seen from the trail-produced component that each rib of it is fully filled. In general, heat treatment is required to ensure the comprehensive mechanical properties of it after forming [21]. Therefore, the subsequent section discusses the effects of different heat treatments on the mechanical properties of the component.

## 4. Heat Treatment and Testing

### 4.1. Design of Heat Treatment Schedule

**Figure 12:**
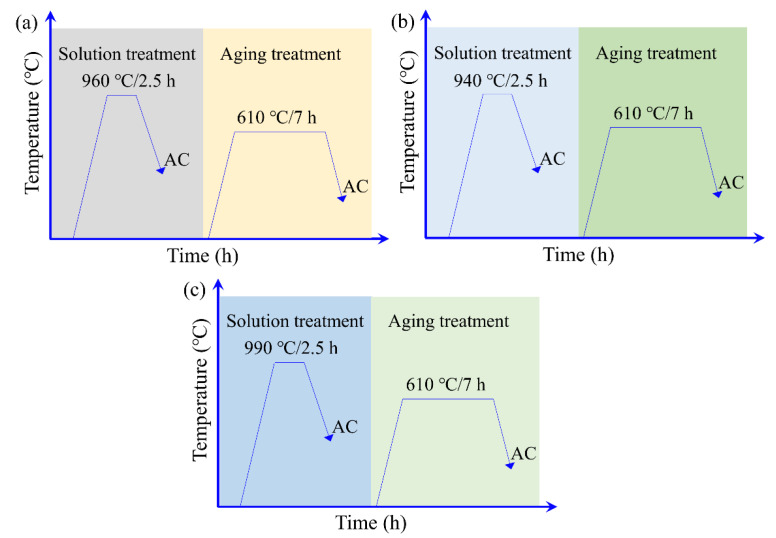
Schematic illustration of heat treatment: (**a**) the first heat treatment, (**b**) the second heat treatment, and (**c**) the third heat treatment.

The fully filled component was heat treated with a combination of solution treatment and aging treatment. The designed heat treatment parameters included the heating temperature and holding time, as shown in Figure 12. The air cooling (AC) was adopted for all heat treatment in this work. Specifically, in the first heat treatment method (1st HTM), as shown in Figure 12a, the component was first subjected to a solution treatment at 960 °C for 2.5 h, followed by air cooling (AC). Thereafter, it was aged at 610 °C for 7 h, followed by air cooling (AC) to room temperature (RT). During the second heat treatment method (2nd HTM), as shown in Figure 12b, the component was first subjected to a solution treatment at 940 °C for 2.5 h, followed by air cooling (AC). Thereafter, it was aged at 610 °C for 7 h, followed by air cooling (AC) to room temperature (RT). In the third heat treatment method (3rd HTM), as shown in Figure 12c, the component was first subjected to a solution treatment at 990 °C for 2.5 h, followed by air cooling (AC). Thereafter, it was aged at 610 °C for 7 h, followed by air cooling (AC) to room temperature (RT).

### 4.2. Experiment Procedure

**Figure 13:**
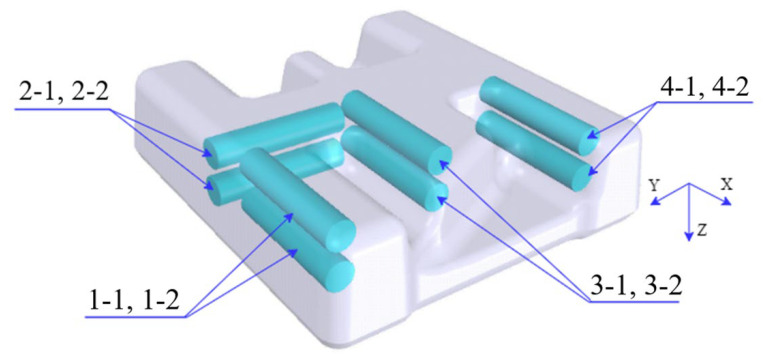
Schematic diagram of the sampling position of mechanical properties.

**Figure 14:**
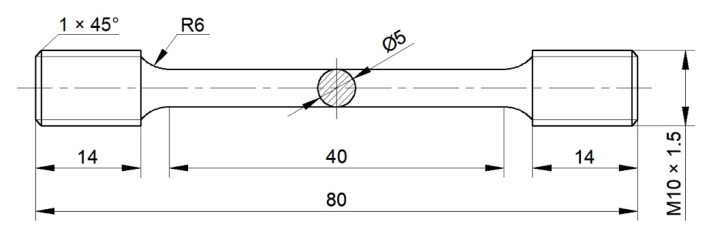
Schematic diagram of tensile specimen.

**Figure 15:**
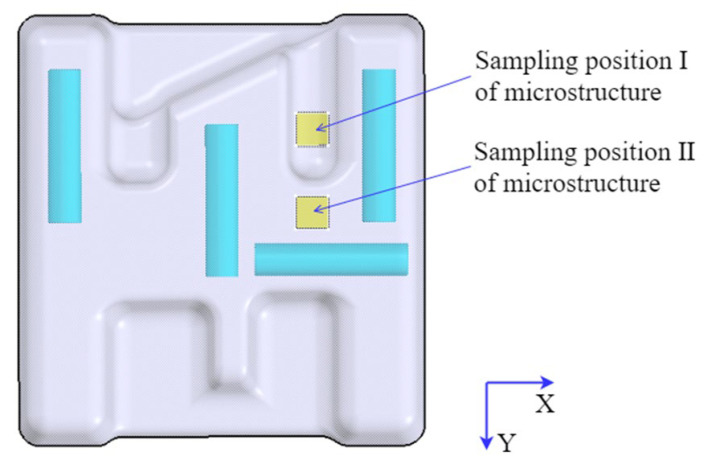
Schematic diagram of the sampling position of microstructure.

The heat treatment of the component was carried out according to the heat treatment schedule in Section 4.1. In order to study the effects of different heat treatment methods on the mechanical properties of the component, the tensile samples were extracted from the component, and the sampling positions are shown in Figure 13. There were four groups of tensile specimens, which were mainly selected from four different positions, and each group had two specimens. The two samples were selected to ensure the accuracy of the results. The specimen numbers of the first, the second, the third, and the fourth group correspond to (1-1, 1-2), (2-1, 2-2), (3-1, 3-2), and (4-1, 4-2), respectively, wherein the first and the second group of specimens were tested for mechanical properties at room temperature, and the third and the fourth group of specimens were tested for mechanical properties at high temperatures. The mechanical properties at high temperatures were mainly carried out at 450 °C and 650 °C. In the assumed coordinate system (in Figure 13), the selected specimens were parallel to the X and Y directions, respectively. The specific dimensions of the tensile specimen are shown in Figure 14. After different heat treatments, the microstructure of the component was observed via a microscope. The positions of the specimen for the microscopic observation are shown in Figure 15.

### 4.3. Microstructural Analysis

**Figure 16:**
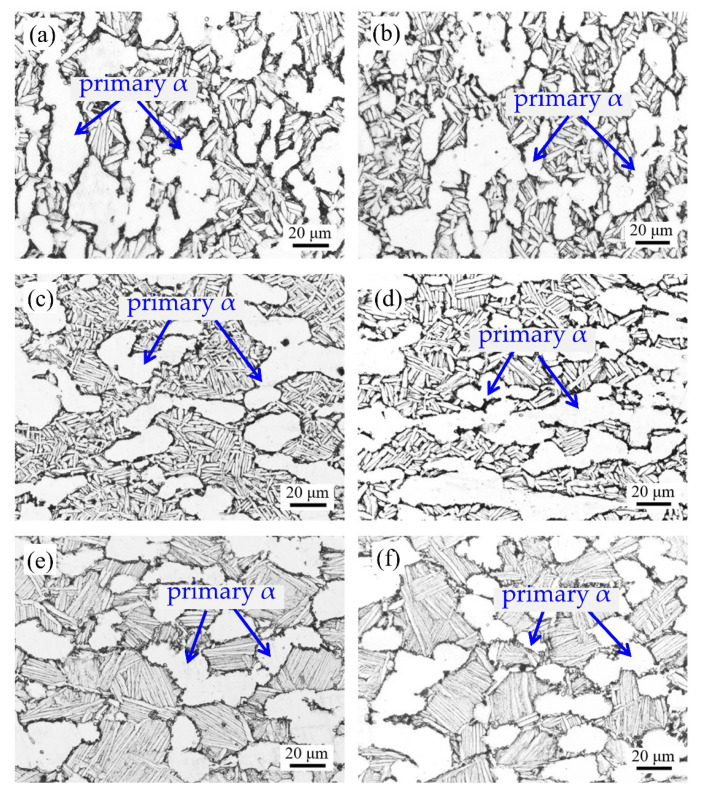
Microstructure of different heat treatments: (**a**,**b**) correspond to the first heat treatment, (**c**,**d**) correspond to the second heat treatment, and (**e**,**f**) correspond to the third heat treatment.

Figure 16 shows the microstructure of different heat treatment methods. The content and shape of the primary α phase in TC31 titanium alloy also change with different heat treatment methods, but the main microstructure is α + β dual phase. As shown in Figure 16a,b, the shape of the primary α phase is mainly elliptical and short rod, which has a certain directionality. The shape of the secondary α phase is thick and needle-like, and its length dimension is about 15 μm. As shown in Figure 16c,d, the primary α phase is mainly an ellipse, an intermittent equiaxed structure. The shape of the secondary α phase is fine and needle-like, and the distribution is longitudinal and transverse, resembling a basketweave structure with a length dimension of about 10 μm [24]. As shown in Figure 16e,f, the primary α phase is mainly a spherical equiaxed structure, and the secondary α phase is a long needle-like structure with a length of about 20~30 μm. From the perspective of a solid solution temperature, for Figure 16, namely from Figure 16c,d to Figure 16a,b, and then to Figure 16e,f, the higher the temperature, the more sufficient the energy for dynamic recrystallization, the better the spheroidization effect of the primary α phase, and the larger the size of the secondary α phase.

### 4.4. Performance Testing

**Figure 17:**
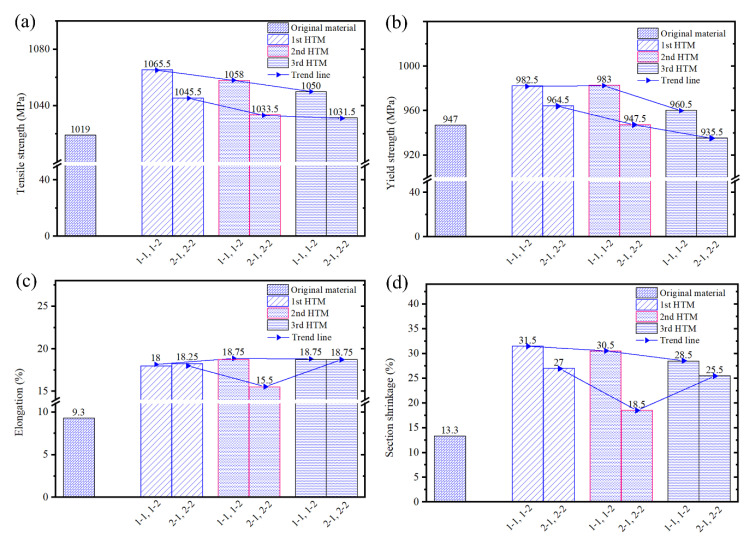
Mechanical properties of tensile specimens at room temperature: (**a**) the tensile strength, (**b**) the yield strength, (**c**) the elongation, and (**d**) the section shrinkage.

Figure 17 shows the mechanical properties of tensile specimens at room temperature. Figure 17a shows the ultimate tensile strength at room temperature. It can be seen from the figure that the ultimate tensile strength of the component after forging and heat treatment has been improved compared to the original material; the first HTM improves the most (46.5 MPa and 26.5 MPa in the X and Y directions, respectively), and the third HTM improves the least. Figure 17b shows the yield strength at room temperature. It can be seen from the figure that the yield strength of the component after forging and heat treatment in the X direction was improved, and the maximum increase was 36 MPa, but the yield strength in the Y direction was slightly decreased (compared to the original material) when the third heat treatment method is adopted. On the whole, the ultimate tensile strength and yield strength were improved compared with that of the original material. All of them show a decreasing tendency from the first HTM to the second HTM, and then to the third HTM.

Figure 17c shows the elongation at room temperature. It can be seen from the figure that the elongation of the component after forging and heat treatment was significantly improved, and the elongation is about twice that of the original material. Figure 17d shows the section shrinkage at room temperature. It can be seen from the figure that the section shrinkage of the component after forging and heat treatment was greatly improved, and the maximum increase was 18.2% after using the first HTM, which is more than once compared with the elongation of the original material.

In a word, from the four aspects, i.e., ultimate tensile strength, yield strength, section shrinkage, and elongation, it can be found that the mechanical properties after the forging and heat treatment were significantly improved. By comparison, the first heat treatment method (the component was first subjected to a solution treatment at 960 °C for 2.5 h, followed by air cooling) has the best effect on the mechanical properties of the component.

**Figure 18:**
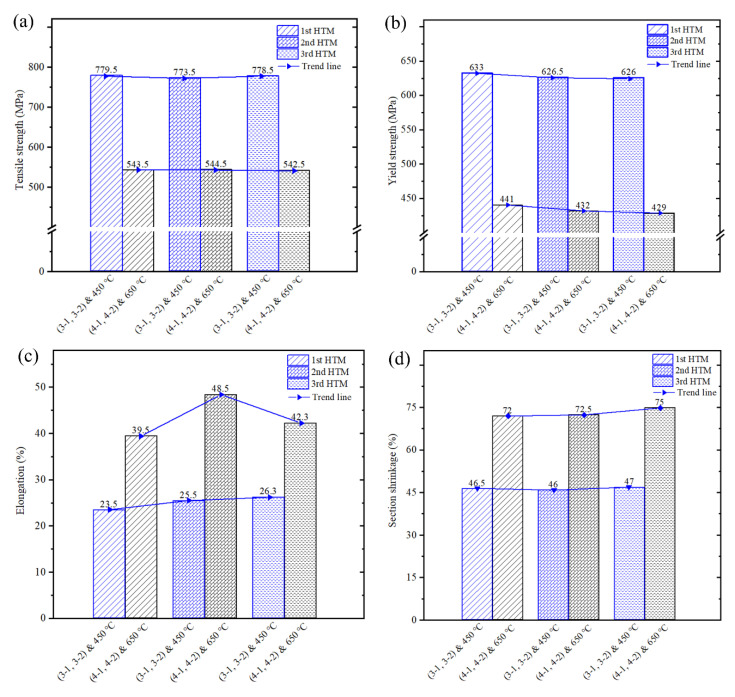
Mechanical properties of tensile specimens at high temperature: (**a**) the tensile strength, (**b**) the yield strength, (**c**) the elongation, and (**d**) the section shrinkage.

Figure 18 shows the mechanical properties of tensile specimens at high temperatures. It can be seen from Table 2 that the ultimate tensile strength of the original material at 450 °C and 650 °C is 767 MPa and 508 MPa, respectively; the yield strengths is 624 MPa and 409 MPa, respectively; the elongation is 21.4% and 33.8%, respectively; and the section shrinkage is 39.3% and 41.3%, respectively.

Figure 18a shows the ultimate tensile strength of the component at high temperatures. They were improved by forging and heat treatment. The tensile strength at 450 °C and 650 °C was increased by about 15 MPa and 35 MPa, respectively, compared with the original material, but the improvement range is not obvious compared with the room temperature, so the effects of different heat treatment methods on high-temperature ultimate tensile strength is not significant. Figure 18b shows the yield strength of component at high temperatures. They were improved by forging and heat treatment. The maximum amplitude of increase at 450 °C and 650 °C corresponds to 9 MPa and 32 MPa, respectively. At the same time, it can be found that different heat treatment methods at high temperatures have no significant effect on the tensile strength and yield strength of component; this is different from that at room temperature.

Figure 18c shows the elongation of component at high temperatures. They were improved after forging and different heat treatments, and the elongation at 650 °C increased significantly compared with 450 °C. Figure 18d shows the section shrinkage of the component at high temperatures. After the forging and heat treatment, the section shrinkage of 450 °C and 650 °C increased by about 7% and 30%, respectively, which shows that different heat treatments can obviously enhance the plasticity (the elongation and section shrinkage) of the component at the temperature of 650 °C. Meanwhile, it can be found that different heat treatment methods have a little effect on the plasticity (the elongation and section shrinkage) at the temperature of 450 °C.

## 5. Conclusions

The forming and heat treatment of the TC31 titanium alloy component with high ribs and thin webs were systematically studied in this paper. Based on the results of these studies, the main conclusions are summarized as follows:

(1) The forming load of the component gradually increases during the pre-forging and final forging processes till the maximum load at the end of forming is 1920 kN.
(2) The folding angle of the component in the forming process is mainly distributed from 180° to 210°. The material flow law of the typical section of the component is revealed, and a fully filled component is obtained through trial production.
(3) The better heat treatment method for the component is to conduct solution treatment for 2.5 h at 960 °C, and then conduct aging treatment for 7 h at 610 °C.
(4) The mechanical properties of the original material were improved by using forging and optimal heat treatment methods. The maximum increases of ultimate tensile strength, yield strength, elongation, and section shrinkage at room temperature are 46.5 MPa, 35.5 MPa, 8.7%, and 18.2%, respectively.
(5) When comparing the high-temperature mechanical properties at 450 °C and at 650 °C, we found that the effect of the heat treatment on improving the mechanical properties at 650 °C is better than that at 450 °C, thus further demonstrating that the designed heat treatment provides a good foundation for satisfying the use of this high-temperature titanium alloys at higher temperatures.

## Figures and Tables

**Table 1:** The composition of high-temperature TC31 titanium alloy (wt%).

Element	Al	Sn	Zr	Nb	Mo	W	Si	Ti
Composition	6.0~7.5	2.5~3.5	2.5~3.5	1.0~3.2	1.0~3.2	0.3~1.2	0.1~0.5	Bal.

**Table 2:** The mechanical properties of TC31 titanium alloy.

Temperature	UTS/MPa	YS/MPa	Elongation/%	Section Shrinkage/%
RM	1019	947	9.3	13.3
450 °C	767	624	21.4	39.3
650 °C	508	409	33.8	41.3

**Table 3:** Parameters of finite element simulation.

Simulation Parameters	Values
Temperature of the billet	975 °C
Temperature of the die	300 °C
Speed of the top die	7 mm/s
Coefficient of friction	0.3
Heat transfer coefficient	5 N/s/mm/°C
Number of mesh	100,000

## Data Availability

Not applicable.
